# An Unusual Presentation of Tumor Lysis Syndrome in a Patient with Advanced Gastric Adenocarcinoma: Case Report and Literature Review

**DOI:** 10.1155/2012/468452

**Published:** 2012-05-27

**Authors:** Danica Maria Vodopivec, Jose Enrique Rubio, Alessia Fornoni, Oliver Lenz

**Affiliations:** Department of Nephrology and Hypertension, Jackson Memorial Hospital, 1611 North West 12th Avenue, Miami, FL 33101, USA

## Abstract

Tumor lysis syndrome (TLS) is characterized by hyperuricemia, hyperkalemia, hyperphosphatemia, and secondary hypocalcemia in patients with a malignancy. When these laboratory abnormalities develop rapidly, clinical complications such as cardiac arrhythmias, acute renal failure, seizures, or death may occur. TLS is caused by rapid release of intracellular contents by dying tumor cells, a condition that is expected to be common in hematologic malignancies. However, TLS rarely occurs with solid tumors, and here we present the second chemotherapy-induced TLS in a patient with advanced gastric adenocarcinoma to be reported in the literature. We also provide information regarding the total cases of TLS in solid tumors reported from 1977 to present day. Our methodology involved identifying key articles from existing reviews of the literature and then using search terms from these citations in MEDLINE to find additional publications. We relied on a literature review published in 2003 by Baeksgaard et al., where they gathered all total 45 cases reported from 1977 to 2003. Then, we looked for new reported cases from 2004 to present day. All reports (case reports, brief reports, letters to editor, correspondence, reviews, journals, and short communications) identified through these searches were reviewed and included.

## 1. Introduction

Tumor lysis syndrome is a life-threatening oncologic emergency that occurs when a large amount of malignant tumor cells breakdown rapidly and release their intracellular contents into the systemic circulation causing electrolyte and metabolic disturbances, such as hyperuricemia, hyperkalemia, hyperphosphatemia, and hypocalcemia. Renal insufficiency, cardiac arrhythmias, seizures, and death due to multiorgan failure may be the final consequences of these biochemical derangements [1–8].

**Figure 1:**
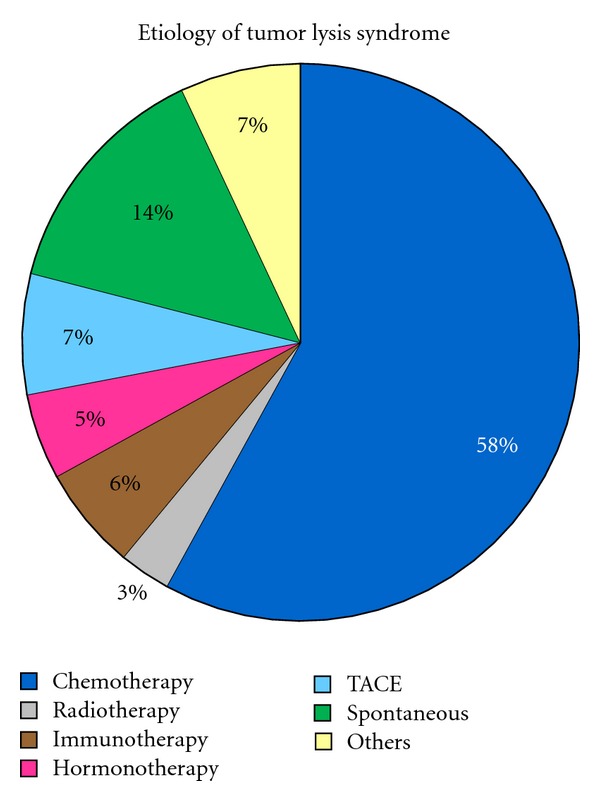
Etiology of tumor lysis syndrome. TACE: transarterial chemoembolization. Others include surgery, bisphosphonates, radiofrequency, combination of different cancer therapies.

**Figure 2:**
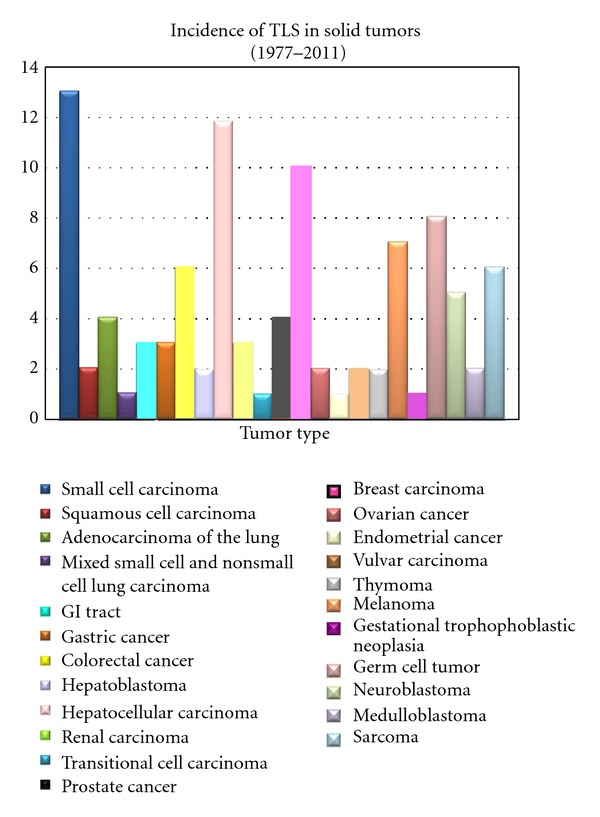
Reported cases of tumor lysis syndrome in solid tumors.

Tumor lysis syndrome develops usually after the initiation of chemotherapy but in rare cases may arise spontaneously before any antitumor therapy has been initiated [1, 4–19] (Table 1) (Figure 1). TLS is common in patients with hematologic malignancies with high growth rates or a large disease burden, but it is rarely observed in patients with solid tumors of which to date there are only 100 cases described in the literature [1–93] (Table 1) (Figure 2).

To the best of our knowledge, the present study case is the second report describing TLS following chemotherapy for advanced gastric adenocarcinoma [37]. Also, a review of the literature regarding TLS in solid tumors is presented and recommendations for management are discussed.

## 2. Case Report

The patient is a 57-year-old Hispanic man with a history of stage III moderately differentiated gastric adenocarcinoma intestinal type, diagnosed in late 2006. He was enrolled in a clinical trial for a new regimen of neoadjuvant chemotherapy consisting of oxaliplatin (85 mg/m² i.v. over 2 hours on days 1 and 15), docetaxel (25 mg/m² i.v. over 30 minutes on days 1, 8, 15), floxuridine (110 mg/kg i.v. over 24 hours on days 1, 8, 15), and leucovorin calcium (500 mg/m² i.v. over 24 hours on days 1, 8, 15) with treatment repeated every 4 weeks. After the second course (January 2007), he underwent resective therapy (partial gastrectomy) followed by 2 more cycles of the above-mentioned chemotherapy regimen as an adjuvant therapy.

Five months later, recurrence of the primary tumor was found in the liver; therefore, the patient was started on a different regimen (paclitaxel 120 mg/m², floxuridine 150 mg/kg, leucovorin 500 mg/m², and cisplatin 100 mg/m²), and liver segmentectomy was performed. 

Unfortunately, patient failed regular controls, and by 2011 a CT of the chest and abdomen revealed extensive metastatic liver nodules which ranged from 3.5 cm to sub-centimeter in size, and metastasis to the sternum were also found. Consequently, he was again included in the initial experimental chemotherapy regimen as a first line therapy for his metastatic disease (oxaliplatin, docetaxel, floxuridine, and leucovorin). 

Seven days after receiving the first chemotherapy cycle, he developed nausea, vomiting, oliguria, generalized weakness, and was referred to the emergency department by his oncologist due to abnormal laboratory data (Table 2). 

On arrival the patient was alert and appeared in poor general condition, pale, volume depleted with low blood pressure (102/63 mmHg), tachycardia (102/min), respiratory rate of 20/min, and body temperature of 36.6°C. A firm, nontender, 2 cm below the costal margin of the right midclavicular line hepatomegaly was appreciated. There was no peripheral lymphadenopathy, and laboratory findings are shown on Table 2. 

**Figure 3:**
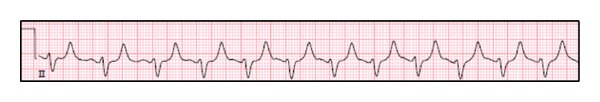
EKG from the patient's admission showing atrial fibrillation with rapid ventricular response and peaked T waves.

Urinalysis determined urine pH of 5.0 (4.5–7.5). The chest radiograph appeared to be normal. An EKG demonstrated atrial fibrillation with rapid ventricular response, left anterior fascicular block, and peaked T waves (Figure 3). A renal ultrasound examination revealed an increased bilateral cortical echogenicity but no evidence of hydronephrosis. 

A diagnosis of chemotherapy-induced TLS with acute renal failure was made. The patient was given vigorous volume expansion, intravenous sodium bicarbonate, calcium gluconate, insulin given together with 50% dextrose, and allopurinol. His laboratory data did not improve and hemodialysis was started. The patient underwent a total of 6 hemodialysis over a course of two weeks and was discharged 18 days after with normal serum electrolyte and metabolic parameters. However, his renal function continued to be impaired with a serum creatinine of 3.86 mg/dL, blood urea nitrogen of 23 mg/dL, and eGFR of 16. 

## 3. Review of the Literature

TLS is an oncologic emergency characterized by severe electrolyte and metabolic abnormalities due to a rapid and massive lysis of malignant cells with the release of intracellular contents into the bloodstream, overwhelming the excretory and reutilization capacities of the body, leading to hyperuricemia, hyperkalemia, hyperphosphatemia, and secondary hypocalcemia [1–8]. As a consequence, these biochemical disorders can become clinically relevant and present as renal insufficiency, cardiac arrhythmias, seizures, and death due to multiorgan failure [1–8]. Cairo and Bishop published the most complete and often referenced definition of TLS in 2004. They classified TLS into two groups, separating patients exhibiting only laboratory findings of TLS from those with potentially life-threatening clinical presentations. Cairo and Bishop definitions of laboratory and clinical TLS are summarized in Table 3 [1–4, 6, 7]. 

In the setting of a malignancy with a high proliferation rate, large tumor burden, and/or high sensitivity to treatment, initiation of anticancer treatment, such as chemotherapy, radiotherapy, resective surgical procedures, immunotherapy, hormonotherapy, radiofrequency ablation, or sometimes glucocorticoid therapy alone may result in the rapid lysis of tumor cells [1, 5–8] (Table 1) (Figure 1). In addition, TLS can occur spontaneously from the tumor necrosis prior the onset of anticancer therapy [1, 4–19] (Table 1) (Figure 1). 

Tumor and host-related factors associated with an increased risk of TLS in solid tumors are mentioned in Table 4 [1–8]. The tumor-related risk factors are common characteristics of hematological malignancies, where TLS is a well-known clinical problem, especially in acute lymphoblastic leukemias and high-grade non-Hodgkin's lymphomas, and particularly in Burkitt's lymphoma [1–7]. However, TLS can rarely occur in solid tumors that have a high proliferative rate, large tumor burden, and high sensitivity to cytotoxic therapy [1, 4, 6]. 

According to the literature review, there have been 100 reported cases of TLS in patients with solid tumors from the first report in 1977 to 2011 (Table 1) (Figure 2), including small cell carcinomas [9, 20–30], squamous cell carcinomas [10, 11], adenocarcinomas of the lung [12, 31–33], mixed small cell and nonsmall cell lung carcinoma [34], gastrointestinal carcinomas [13–15, 35–42], hepatoblastomas [43, 44], hepatocellular carcinomas [15, 45–53], renal carcinomas [54–56], transitional cell carcinoma [57], prostate carcinomas [58–61], breast carcinomas [16, 30, 62–68], ovarian carcinomas [69, 70], endometrial carcinoma [71], vulva carcinomas [72, 73], thymomas [74, 75], melanomas [76–82], gestational trophoblastic neoplasia [83], germ cell tumors [17, 18, 64, 84, 85], neuroblastomas [86, 87], medulloblastomas [88, 89], and sarcomas [19, 90–93]. Most cases regarded as TLS in solid tumors were chemotherapy-induced, even though various other causes were pointed out for TLS in solid tumors as well [3] (Table 1) (Figure 1). The dominant factor for developing TLS in solid tumors, which occurred in all 100 cases, was having a large tumor burden. Another relevant fact was the presence of metastatic disease observed in 83% of all cases, of which the liver was the most affected organ; thus, the remaining 17% presented with bulky primary tumor. 

Other risk factors for developing TLS in solid tumors included pretreatment increased LDH, renal impairment, hyperuricemia, and hyperphosphatemia found in 86% (43/50), 26% (18/69), 42% (21/50), and 14% (6/44) of the evaluable patients in which specific results were reported, respectively. 

In 7% of the cases, we found hydronephrosis as a high risk for developing TLS due to compression of the genitourinary tract by the tumor, being particular hazard neoplasms such as germ cell tumor, prostate cancer, retroperitoneal sarcoma, and uterus carcinoma. 

Irrespective of the cancer type, there is a 20–50% increase in mortality for undiagnosed or late-diagnosed TLS cases in solid tumors [8]. This high mortality rate may be due to the fact that in solid tumors, contrary to what is observed in hematological malignancies, TLS is usually evident after a few days within treatment when the patient may have already left the hospital [3, 8]. Another cause is that in hematological malignancies, prophylactic measures are more often implemented, and awareness of the syndrome is higher. The fatality rate of the 100 cases presenting TLS in solid tumors was found to be 41%. 

Symptoms associated with TLS strongly reveal electrolyte and metabolic irregularities, which are hyperkalemia, hyperphosphatemia, secondary hypocalcemia, and hyperuricemia [1–8]. Hyperkalemia, which takes place within 12 to 24 hours from the antineoplastic treatment's initiation, becomes the earliest and most severe laboratory finding [3]. The clinical manifestations for hyperkalemia include lethargy, weakness, paresthesias, muscle cramps, nausea, vomiting, and abnormalities in the electrocardiogram, such as peaked T waves, prolonged PR interval, widened QRS complexes, ventricular tachycardia, ventricular fibrillation, or asystole [2–5]. Since the phosphorus concentration is higher in malignant cells than in normal cells (4 : 1), accelerated tumor breakdown usually leads to hyperphosphatemia with the subsequent secondary hypocalcemia [1, 3–7]. Both electrolyte disturbances, hyperphosphatemia and hypocalcemia, may cause a variety of symptoms, such as nausea, vomiting, lethargy, muscle cramps, tetany, seizures, cardiac arrhythmias, and/or acute renal failure [2–6]. Accumulation of uric acid may increase the urinary uric acid concentration to a point where precipitation occurs, and acute renal failure may be the consequence. The widespread use of agents lowering uric acid production or excretion is responsible for nephrocalcinosis, becoming a major mechanism of acute renal failure in TLS [1–3, 6]. However, calcium phosphate precipitation does not only occur in the kidneys, but also in other soft tissues such as the heart, where it may induce cardiac arrhythmias [1–3, 6]. 

The fact that urine is acidic facilitates uric acid to precipitate, thus excessive overproduction and over excretion could accumulate uric acid in the form of crystal precipitation in the renal tubules, leading to obstructive uropathy with acute kidney injury [1, 2, 4, 5, 8, 65]. Uric acid or calcium phosphate accumulation does not always imply urinary tract symptoms; however, flank pain due to renal pelvic or ureteral stone formation may occur [1, 4]. The urinalysis quite often discloses a number of uric acid crystals, amorphous urates, calcium phosphate crystals, and/or hematuria [1, 4]. 

The treatment principles for TLS are prevention, early diagnosis, and proper management of disorders [3]. In 2008, an international expert panel published evidence-based guidelines, for the prevention and treatment of TLS depending on the malignancy risk category. According to this evidence-based guidelines unless tumor and/or host-related risk factors are associated, solid tumors are considered low risk (<1%) for developing TLS. Thus, adequate recommendations for the prevention and management of TLS in solid tumors are accurate hydration prior to therapy and a “watch and wait” approach with close monitoring on looking for the first biologic derangements characterizing TLS (hyperuricemia, hyperphosphatemia, hypocalcemia, increased creatinine level) without any form of prophylactic hypouricemic therapy or phosphate binders [1–3, 6, 37]. 

Once TLS is established, the standard therapy strategy for treating TLS is based on volume expansion, decreasing the metabolic abnormalities, and in most cases providing supportive treatment of renal failure [1, 5, 8]. 

Administration of fluids is important because it increases renal blood flow, glomerular filtration, and reduces the urinary supersaturation of uric acid, calcium, and phosphate [2, 4, 5, 7]. Patients should receive 3 l/m²/day (200 mL/Kg/day if ≤10 kg) and have a urine output ≥100 mL/m²/h (3 mL/kg/h if ≤10 kg) [2–4, 6]. In the absence of obstructive uropathy and/or hypovolemia, diuretics may be used to maintain the adequate urine output [2–4, 6, 7]. The utility of low-dose dopamine to enhance renal blood flow is not as clear [3]. 

Maintaining urine pH above 7 with the aid of acetazolamide and/or sodium bicarbonate for the prevention and/or treatment of TLS may reduce the precipitation of uric acid crystals but at the same time may decrease the solubility of calcium phosphate or xanthine, and in the setting of acute renal failure, metabolic alkalosis may be an unintended consequence [1, 3, 4, 6, 7]. Consequently, the use of sodium bicarbonate should be restricted to those patients with severe metabolic acidosis [1]. 

Hyperkalemia may be an acute life-threatening emergency in patients with TLS. In case of hyperkalemia, cardiac membrane stabilization with 10% calcium gluconate may not be effective in the setting of severe hyperphosphatemia, and given an increased risk of calcium phosphate tissue precipitation, calcium administration is only appropriate for symptomatic hypocalcemia (arrhythmia, hypotension, tetany, or muscle cramps) [1, 7]. Consequently, measures to reduce serum potassium or enhance potassium excretion should have priority. These include primarily insulin and beta-2 agonists to shift potassium into cells and volume expansion and loop diuretics to enhance potassium elimination. Sodium bicarbonate may be used as a slow continuous infusion in the face of severe acidosis. Exchange resins may be administered but should not be relied upon in the setting of acute hyperkalemia due to their unpredictable efficacy. For patients with renal failure, dialysis should be initiated as soon as possible [1–7]. For hyperphosphatemia, phosphate binders and/or hypertonic dextrose with insulin may be used [1–7]. 

**Figure 4:**
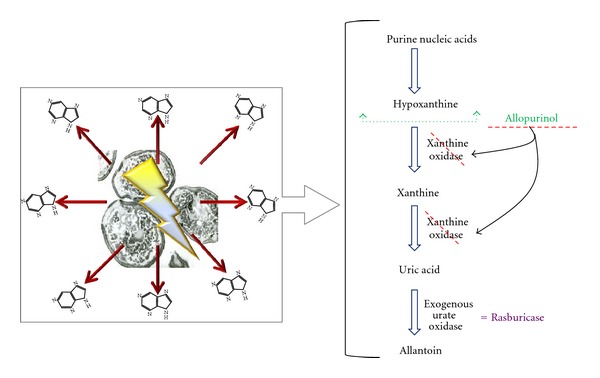
Mechanism of action of hypouricemic agents. Hyperuricemia is a consequence of the catabolism of purine nucleic acids to hypoxanthine and xanthine and then to uric acid via the enzyme xanthine oxidase. Allopurinol is a competitive inhibitor of the enzyme xanthine oxydase. Rasburicase (exogenous urate oxidase) leads uric acid to a more soluble compound, allantoin.

Allopurinol is a competitive inhibitor of xanthine oxidase (Figure 4), and it has been successfully prescribed for the treatment of hyperuricemia. Allopurinol is metabolized to oxypurinol, which in turn inhibits xanthine oxidase; therefore, it blocks the metabolic process of xanthine and hypoxanthine to uric acid, leading to an increase in the levels of these two metabolites [1, 3–6]. As a result, patients with massive TLS who have been prescribed allopurinol may develop xanthine precipitation leading to acute renal failure [1, 2, 4, 5, 7, 65]. This can be avoided by replacing allopurinol with urate oxidase, which catabolizes uric acid to the more soluble compound allantoin [1–6] (Figure 4). Urate oxidase is an enzyme that can be found in many mammals except humans [1, 3, 4, 6]. The two available commercial forms of urate oxidase are a nonrecombinant form (uricozyme) and a recombinant form (rasburicase), which is the preferred one. Rasburicase has been shown to be more effective than allopurinol, since it has higher uricolytic effect [1, 3–7], it does not increase xanthine levels [1, 2, 4, 5, 7], and it reduces high uric acid levels prior treatment initiation [1–7]. 

In some patients, despite prophylactic and therapeutic measures, acute renal failure ensues, and its management includes careful monitoring of fluid intake and output, electrolyte balance, hypertension control [4], and renal dialysis [94]. Indications for renal replacement therapy include volume overload with pulmonary edema or hypertension that is refractory to therapy, low urine output (oliguria), persistent or symptomatic hyperkalemia, hyperphosphatemia, hypocalcemia, and/or hyperuricemia despite conservative measures, metabolic acidosis with pH less than 7.2 or bicarbonate less than 10 mEq/l, rapidly rising blood urea nitrogen greater than 150 mg/dL, and neurologic symptoms secondary to uremia or electrolyte imbalance [4, 95]. From the above-mentioned criteria, oliguria and other biochemical disturbances related to TLS have become more frequent indications for dialysis than hyperuricemia since the introduction of rasburicase [2, 94]. Both hemodialysis and hemofiltration are affective, while peritoneal dialysis is not [94, 96]. Dialysis should be continued until proper renal function is reestablished.

## 4. Discussion

Our patient with metastatic gastric adenocarcinoma developed metabolic (hyperkalemia, hyperuricemia, hyperphosphatemia, and hypocalcemia) and clinical (cardiac arrhythmia and acute renal failure) derangements 7 days after instituting chemotherapy, meeting CTLS diagnostic criteria. According to previous reports, there are 2 patients with advanced gastric adenocarcinomas who developed TLS documented in the literature (34, 35), although only one occurred after chemotherapy [37] (Table 5). Therefore, our case would be the second gastric adenocarcinoma chemotherapy-induced TLS but the first to be reported after the use of the experimental chemotherapeutic regimen consisting on oxaliplatin, leucovorin, floxuridine, and docetaxel. 

Chemotherapy-related nephrotoxicity was less likely due to the fact that the anticancer agents the patient was receiving are not considered to be nephrotoxic, except for oxaliplatin and docetaxel which have less than a 1% probability of causing renal failure, and it usually occurs with high doses and after repeated exposures. 

Large tumor burden, multiple metastases to liver and bone, the use of combination chemotherapy drugs, pretreatment elevated LDH, and dehydration were the risk factors that placed the patient in a high-risk group for developing TLS [1–8], although his renal function and uric acid levels were normal before getting anticancer treatment. 

The purpose for giving sodium bicarbonate to the patient was to correct the metabolic acidosis, rather than alkalinizing urine. Despite intensive medical treatment, the patient developed refractory electrolyte imbalances, metabolic acidosis, uremia, and oliguria. Thus, hemodialysis was performed with an improvement of the mentioned abnormalities, and the TLS was resolved. However, his renal function never returned to baseline, which has been linked to increased morbidity and mortality. 

Even though solid tumors with high sensitivty to therapy are considered to be at high risk for developing TLS, we noticed that the syndrome was also present in tumor types considered as relatively insensitive to therapy such as melanoma, sarcoma, hepatocellular carcinoma, vulvar carcinoma, non-small cell lung cancer, and gastric cancer. According to our observations, the predominant risk factors to induce TLS in solid tumors are large tumor burden and liver metastases, rather than tumor sensitivity to therapy agents. 

Liver metastases in solid tumors were found in most TLS cases, regardless whether patients had liver function abnormalities or not [3]. Apparently, liver involvement creates a risk for the development of TLS in solid malignancies, possible causes being high purine pools, increased tumor burden, and/or impaired uric acid metabolism [3].

## 5. Conclusion

Even though TLS in solid tumors is a rare condition, physicians should be aware of it, especially in therapy-sensitive large burden tumors with metastatic disease, increased pretreatment LDH, renal impairment, hyperuricemia, and hyperphosphatemia, all of which are here documented to be mayor risk factors for the development of TLS. 

If there is a possibility to develop TLS, the metabolic abnormalities should always be corrected before starting an anticancer regimen, since prevention is the best way to reduce its high morbidity and mortality. 

In conclusion, TLS in solid tumors requires special attention for its prevention, early diagnosis, and management due to its poor clinical course. Physicians should be alert since improvement in the anticancer treatment may increase the incidence of TLS in solid tumors.

## Figures and Tables

**Table 1:** Reported cases of tumor lysis syndrome in solid tumors (1977–2011).

Tumor type	Treatment	Outcome of TLS	Year published	Reference
Small cell carcinoma	DOXO, CDDP, VP-16, VCR	Died	1983	[20]*
	DOXO, CTX, VCR	Resolved	1983	[21]*
	CCNU, CTX, MTX	Died	1988	[22]*
	CDDP, VP-16	Resolved	1988	[22]^†^
	DOXO, CTX, VCR	Resolved	1990	[23]*
	DOXO, IF	Resolved	1991	[24]^‡^
	CDDP, VP-16	Resolved	1997	[25]*
	CDDP, VP-16	Resolved	1997	[26]*
	CDDP, VP-16	Died	1999	[27]*
	CDDP, VP-16	Died	2001	[28]*
	TOPO	Died	2002	[29]*
	CBCDA, VP-16	Resolved	2005	[30]*
	None	Died	2011	[9]*

Squamous cell carcinoma	None	Resolved	2009	[10]^#^
	None	Died	2009	[11]^##^

Adenocarcinoma of the lung	CPT-11, CDDP	Resolved	1998	[31]
	None	Died	2000	[12]
	ZOL	Died	2005	[32]
	DTX	Died	2006	[33]

Mixed small cell and nonsmall cell tumor of the lung	CBCDA, PTX	Died	2002	[34]

GI tract	None	Died	1997	[13]
	SUN	Resolved	2007	[35]
	IMA	Died	2007	[36]

Gastric cancer	None	Resolved	2001	[14]
	CAP, CDDP	Resolved	2008	[37]
	OX, LV, FUDR, DTX	Resolved	2011	Current case

Colorectal cancer	CPT-11	Died	1996	[38]
	CPT-11	Died	2000	[39]
	None	Resolved	2003	[15]
	5-FU, LV, CPT-11	Died	2004	[40]
	CE	Died	2008	[41]
	CPT-11, 5-FU, F, BEV	Died	2008	[42]

Hepatoblastoma	S	Died	1990	[43]
	CDDP, VCR, 5-FU	Resolved	2010	[44]

Hepatocellular carcinoma	TACE	Died	1998	[45]
	TACE	Resolved	1998	[45]
	None	Died	2003	[15]
	RF	Resolved	2005	[46]
	TH	Died	2006	[47]
	TOCE	Died	2007	[48]
	TACE	Resolved	2008	[49]
	TACE	Resolved	2009	[50]
	TACE	Resolved	2009	[50]
	SOR	Died	2009	[51]
	SOR	Resolved	2010	[52]
	SOR	Resolved	2010	[53]

Renal carcinoma	SUN	Resolved	2007	[54]
	SUN	Resolved	2010	[55]
	SUN	??	2011	[56]

Transitional cell carcinoma	GC	Died	2007	[57]

Prostate cancer	DTX	Died	2004	[58]
	CAB	Died	2004	[59]
	PTX	Resolved	2005	[60]
	CAB	Died	2007	[61]

Breast carcinoma	TX	Resolved	1986	[62]
	5-FU, DOXO, CTX	Died	1987	[63]
	CTX, MTX, 5-FU	Resolved	1989	[64]
	MIT	Resolved	1994	[65]
	None	Died	1995	[16]
	PTX	Died	1997	[66]
	RT	Died	2000	[67]
	CAP	Died	2004	[68]
	5-FU, EPR, CTX	Resolved	2005	[30]
	GC, CDDP	Resolved	2005	[30]

Ovarian cancer	CBCDA, CTX	Resolved	1993	[69]
	TOPO	Resolved	2005	[70]

Endometrial cancer	CBCDA, PTX	Died	2010	[71]

Vulvar carcinoma	CDDP, 5-FU	Resolved	1993	[72]
	CDDP, 5-FU	Died	1998	[73]

Thymoma	CDDP, DOXO, CS	Resolved	1997	[74]
	S	Resolved	2004	[75]

Melanoma	TNF-alpha, mAb	Died	1994	[76]
	IL-1, IF-alpha, CDDP. VIN, DTIC	Resolved	1999	[77]
	CDDP, DTIC, IF-alpha	Died	2001	[78]
	CS	Resolved	2002	[79]
	CDDP, VIN, DTIC, IF-alpha, IL-2	Resolved	2004	[80]
	TAI-CDDP	Resolved	2009	[81]
	CS	Died	2009	[82]

Gestational trophoblastic neoplasia	VP-16, MTX, DACT, CTX, VCR	Resolved	2010	[83]

Germ cell tumor	VIN, BL	Resolved	1989	[64]
	CDDP, VP-16, BL	Resolved	2000	[84]
	None	Resolved	2001	[17]
	None	Resolved	2001	[17]
	BL, VP-16, CDDP	Died	2008	[85]
	VP-16, CBCDA	Resolved	2008	[85]
	VP-16, CBCDA	Died	2008	[85]
	None	Died	2010	[18]

Neuroblastoma	VCR, TN, RT	Resolved	1994	[86]
	RT	Resolved	1994	[86]
	VCR, TN, RT	Resolved	1994	[86]
	CTX, TN	Resolved	1994	[86]
	CTX, DOXO, VCR	Resolved	2003	[87]

Medulloblastoma	RT	Resolved	1984	[88]
	CDDP, VP-16	Resolved	2003	[89]

Sarcoma	CTX, ALT	Resolved	1993	[90]
	CBCDA, EPR, VCR	Resolved	1993	[91]
	CDDP, A, DTIC	Resolved	2009	[92]
	None	Resolved	2010	[19]
	None	Resolved	2010	[19]
	VCR, ACT-D, CTX	Resolved	2011	[93]

Total		D: 41; R: 58; ??: 1	100	

5-fluoracilo (5-FU), tumor necrosis factor alpha (TNF-alpha), interferon-alpha (IF-alpha), anti-GD3 ganglioside monoclonal antibody (mAb), transarterial chemoembolization (TACE), transarterial oil chemoembolization (TOCE), autolymphocyte therapy (ALT), combined androgen blockade (CAB), Doxorubicin (DOXO), cisplatin (CDDP), etoposide (VP-16), vincristine (VCR), cyclophosphamide (CTX), lomustine (CCNU), methotrexate (MTX), ifosfamide (IF), topotecan (TOPO), carboplatin (CBCDA), paclitaxel (PTX), zoledronic acid (ZOL), vinblastine (VIN), bleomycin (BL), teniposide (TN), radiotherapy (RT), surgery (S), 5-fluoracilo (5-FU), tamoxifen (TX), mitoxantrone (MIT), capecitabine (CAP), gemcitabine (GC), irinotecan (CPT-11), docetaxel (DTX), corticosteroids (CS), sunitinib (SUN), imatinib (IMA), cetuximab (CE), folinic acid (F), bevacizumab (BEV), dacarbazine (DTIC), oxaliplatin (OX), floxuridine (FUDR), leucovorin (LV), interleukin-1 (IL-1), interleukin-2 (IL-2), transcatheter arterial infusion of cisplatin (TAI-CDDP), radiofrequency (RF), thalidomide (TH), sorafenib (SOR), adriamycin (A), actinomycin-D (ACT-D), dactinomycin (DACT), epirubicin (EPR), died (D), resolved (R), inaccessible data (??), small cell carcinoma: lung (*), colon (^†^), skin (^‡^); squamous cell carcinoma: lung (^#^), maxillary sinus (^##^); none= TLS developed spontaneously. References: [9–93].

**Table 2:** Laboratory values before and after chemotherapy.

Parameters	Normal ranges	Patient's baseline	Abnormal data for which patient was referred to ER*	At ER*
Leukocytes (/mm³)	4,500–11,000	12,000	6,500	7,400
Hemoglobin (g/dL)	14–18	11	8.6	9.6
Hematocrit (%)	42–52	34	26.9	30
Platelets (/mm³)	150,000–400,000	327,000	39,000	37,000
Glucose (mg/dL)	74–106	86	95	104
Sodium (mEq/L)	135–147	138	130	127
Potassium (mEq/L)	3.5–5	4	8.4	8.7
Phosphorus (mg/dL)	3.0–4.5	—	—	13.9
Calcium (mg/dL)	8.4–10.2	8.4	5.4	5.4
Uric acid (mg/dL)	3.0–8.2	4.3	17.8	17.6
BUN (mg/dL)	9–20	18	175	183
Creatinine (mg/dL)	0.8–1.5	1.00	15.4	14.98
Total bilirubin (mg/dL)	0.2–1.3	0.5	0.8	0.7
Alkaline phosphatase (U/L)	38–126	381	235	254
AST/ALT (U/L)	15–46/21–72	48/30	44/53	46/58
eGFR (mL/min)	>60	>60	3	3
LDH (U/L)	0–250	9,027	—	—
pH	7.35–7.45	—	—	7.17
PaCO2 (mmHg)	33–44	—	—	28
Bicarbonate (mEq/L)	22–28	—	—	10

BUN: blood urea nitrogen; AST: aspartate aminotransferase; ALT: alanine aminotransferase; LDH: lactate dehydrogenase. *7 days after chemotherapy.

**Table 3:** Classification of tumor lysis syndrome by Cairo and Bishop.

Laboratory TLS (LTLS)	Uric acid ≥8.0 mg/dL	Phosphorus ≥4.5 mg/dL	Potassium ≥6.0 mmol/L	Calcium ≤7.0 mg/dL or ionized calcium <1.12
Clinical TLS (CTLS)	Acute renal failure	Cardiac arrhythmia	Seizure	Sudden death

References: [1–4, 6, 7].

**Table 4:** Risk factors for tumor lysis syndrome is solid tumors.

Tumor-related factors			Host-related factors
Tumor extension	Cell lysis potential	Pretreatment laboratory findings	(i) Low urinary flow (ii) Dehydration and/or inadequate hydration (iii) Preexisting nephropathy* (iv) Exposure to nephrotoxins (v) Hypotension (vi) Obstructive uropathy
(i) Large tumor burden (ii) Bulky tumor (≥10 cm) (iii) Extensive metastasis ^∧^ (iv) Extrinsic compression of the genitourinary tract by the tumor	(i) High proliferative rate (ii) High sensitivity to anticancer therapy (iii)Type and intensity of initial anticancer therapy (using a combination of multiple chemotherapists)	(i) Elevated LDH (ii) Elevated serum creatinine (iii) Elevated serum uric acid (iv) Elevated serum phosphate	

LDH: lactate dehydrogenase. ^∧^hepatomegaly, splenomegaly, and nephromegaly due to metastasis. Bone marrow infiltration. *A patient with preexisting nephropathy from hypertension, diabetes, gout, or other causes is at greater risk for developing acute kidney injury and TLS. References: [1–8].

**Table 5:** Similarities and differences between chemotherapy-induced TLS in advanced gastric adenocarcinoma reported in 2008 and our case.

Similarities	Differences		
(i) Massive liver metastasis (ii) Elevated pretreatment LDH (iii) Large tumor burden -Outcome of TLS: resolved	Characteristics	2008 case	Actual case
	Histology	Poorly differentiated	Moderately differentiated
	Tumor extension	Primary bulky tumor is present	Primary tumor was not present (previously resected)
	Pretreatment laboratory data	Slightly elevated serum uric acid	—
		Elevated serum phosphate	
	Host-related risk factors for developing TLS	—	Dehydration
	Chemotherapy regimen	Capecitabine and cisplatin	Oxaliplatin, leucovorin, floxuridine, and docetaxel
	Onset of TLS after receiving chemotherapy	3 days	7 days

Reference: [37].
